# Perceived stress among 20-21 year-olds and their future labour market participation – an eight-year follow-up study

**DOI:** 10.1186/s12889-017-4179-x

**Published:** 2017-03-31

**Authors:** Nanna Trolle, Thomas Lund, Trine Nohr Winding, Merete Labriola

**Affiliations:** 1 DEFACTUM, Central Denmark Region, P.P. Ørumsvej 9-11, 8000 Aarhus, Denmark; 2 Danish Ramazzini Center, Occupational Medicine Regional, Hospital Herning, Gl. Landevej 61, 7400 Herning, Denmark; 3 DEFACTUM, Central Denmark Region, Olof Palmes Allé 15, 8200 Aarhus, Denmark; 4 Department of Public Health, Section of Clinical Social Medicine and Rehabilitation, Aarhus University, Nordre Ringgade 1, 8000 Aarhus C, Denmark

**Keywords:** Register data, Kohort, Health, Young adults, Gender difference

## Abstract

**Background:**

Labour market participation among young adults is essential for their future socioeconomic status and health. The aim of this study was to investigate the association between perceived stress among 20–21 year-olds and their labour market participation 8 years later as well as investigate any potential gender differences.

**Methods:**

A cohort of 1640 young adults born in 1983 completed a questionnaire in 2004 in which perceived stress was measured. The cohort was followed in a register of social benefits for 12 months in 2011–2012 and was categorized into active and passive labour market participation. Logistic regression was used to analyse the association between perceived stress and future labour market participation, taking into account effects of potential confounders. The analyses were stratified by gender.

**Results:**

The effects of perceived stress on future labour market participation differed significantly among young women and young men (*p* = 0.029). For young men, higher levels of perceived stress reduced the risk of future passive labour market participation, when adjusting for socioeconomic factors, self-rated health and copings strategies (*p* = 0.045). For young women, higher levels of perceived stress increased the risk of future passive labour market participation, when adjusting for the same potential confounding factors, although unlike the men, this association was not statistically significant (*p* = 0.335).

**Conclusion:**

The observed gender difference has important implications from a public health point of view. Healthcare professionals might need to differentiate between the genders in terms of health communication, research and when developing preventive strategies.

## Background

Labour market participation among young adults is crucial for their future socioeconomic status, working life, sense of social identity, health and well-being [1–3].

Furthermore, labour market participation among young adults is important to the society, especially Western societies facing persistent youth unemployment and an ageing labour force. If young adults do not participate in the labour market, the society is deprived of labour, leading to increased public expenditures in terms of social benefits [2]. Labour market participation in young adulthood is therefore of major interest within Western countries [2].

In order to reduce individual and societal costs, and to ensure that future demands for labour will be met, it is important to investigate factors and mechanisms associated with lack of participation in the labour market.

Previous studies have shown that factors present early in life can have substantial impact on labour market participation later in life [4–7].

A Danish longitudinal study from 2013 found that negative life events before age 14–15 increased the risk of receiving social benefits at the age of 21–22, especially among girls [4]. An American longitudinal study from 2013 found that young people with depressive symptoms had a higher risk of less employment and lower income later in life [6]. A Swedish study including nearly all Swedish men born between 1950 and 1970 showed that the health of the young men had long-term effects on the future labour market performance, and that the strongest negative effects were due to psychological illnesses [5].

Recently there has been an increased awareness that perceived stress among young adults is related to both mental and physical health [8–11]. There is persuasive evidence that the experience of stress among young adults is related to a poorer mental health, including depression and suicide attempts [8, 10, 12]. A high level of perceived stress is also related to poor self-rated health and an unhealthy lifestyle [8, 9, 13]. Young women typically report significant higher levels of perceived stress than young men [13–15] and there is evidence suggesting that women respond to and handle stress differently than their male counterparts [16, 17].

Due to the fact that perceived stress in young adulthood is related to an unhealthy lifestyle as well as poor health and well-being, it is likely that perceived stress also affects future labour market participation. To the knowledge of the authors, no study has yet examined this potential association.

The aim of this study was therefore to examine the association between perceived stress in young adulthood and the future labour market participation as well as investigate any potential gender differences.

## Methods

### Data and population

The data was taken from a questionnaire survey – the West Jutland Cohort Study [4, 14], consisting of a cohort of young adults born in 1983 and living in the county of Ringkøbing, Denmark, in the spring of 2004.

Initial data was collected in April 2004, when the participants were 20–21 years old.

**Fig. 1:**
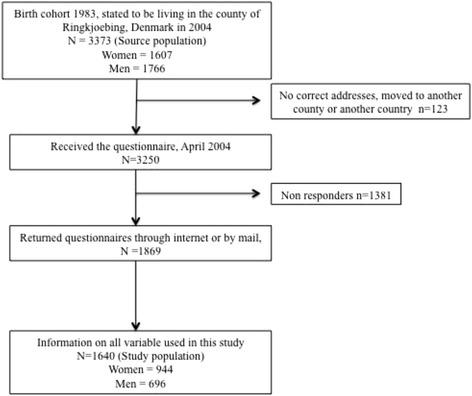
Flowchart of the participants in the study

The source population comprised 3373 individuals, of which 123 were excluded due to missing addresses or because they had moved to another county or another country. A total of 3250 received the initial questionnaire, of whom1869 participated and 1640 answered all questions used in this study (see the flowchart, Fig. 1). The study population of 1640 individuals thus correspond to a total response rate of 49%, 59% for women and 39% for men.

### Outcome

Data on labour market participation (LMP) was obtained from the DREAM register. DREAM is a national register of all public transfer payments from mid-1991 to date. DREAM includes transfers in relation to state educational grants, unemployment benefits, sickness absence compensation, disability pension, immigration and death [18].

LMP was defined according to the amount of social benefits received in a 52-week period from week nine in year 2011 to week eight in year 2012, when the participants were 27–29 years old.

LMP was divided into two categories; *active* and *passive*, depending on the amount of received social benefits. *Active* LMP includes participants who did not receive any social benefits, those who received maternity leave benefits, senior trainee benefits or state educational grants. *Passive* LMP includes participants who received any other benefits. These were either health related benefits (sickness absence compensation, vocational rehabilitation benefits, permanent disability benefits) or unemployment benefits of any sort.

### Exposure

Data on perceived stress was obtained from the questionnaire survey in 2004. The information was merged with the information on social benefits using a unique identifier for each participating individual.

Perceived stress was assessed using a Danish four item version of *the Perceived Stress Scale* (PSS), developed by Cohen et al. and based on Lazarus’s cognitive stress model [19]. According to Lazarus (and Folkman), perceived stress is “(…) a particular relationship between the person and the environment that is appraised by the person as taxing or exceeding his or her resources and endangering his or her well-being” [20].

PSS is a global measurement tool, which is simple to use, and studies have confirmed the reliability and validity of the scale [19, 21].

The four items were: In the last month, how often have you felt; 1) no influence on the essential things in your life? 2) confident about your ability to handle your personal problems? 3) that things were going your way? 4) difficulties were piling up so high that you could not overcome them?

For each item, the participants could score between zero and four points. Item 1) and 4) were scored; *never = 0, almost never =1, occasionally = 2, often = 3* and *very often =4*. Item 2) and 3) were scored reversed. The total scale ranged from 0 to 16 points.

PSS has no clinical cut points, but with regards to the interpretation of the study the perceived stress was divided into three levels according to the number of points. *Low* level of perceived stress was defined as a score between zero and four points, *medium* level between five and nine points and *high* level between 10 and 16 points.

Perceived stress was categorised in order to get a more nuanced picture of the meaning of stress and to have the opportunity to study a possible dose-response relationship.

#### Potential confounders

##### Demographic factors

Data on gender and ethnicity was obtained from DREAM. Ethnicity was divided into three categories: *Danish, Western* and *non-Western*. Due to few numbers in the Western and non-Western category, ethnicity was not included in the analysis.

### Socioeconomic (SE) factors

#### Household (parents’) income

Information on household income was obtained from Statistics Denmark, from the year of 1997 [22]. The variable was divided into three categories: *lowest* (0–36.913 EUR), *medium* (36.913–74.059 EUR) and *highest* (>74.059 EUR) income. The cut points were defined after the tertiles of the household income distribution in the source population.

#### Secondary education completion in 2004

Data on secondary education completion was collected from Statistics Denmark [23], and the variable was divided into two categories; *completed secondary education* and *not completed secondary education*.

#### LMP in 2004

The variable was constructed in the same way as the outcome variable. The information was gathered from week 18 in 2003 to week 17 in 2004.

#### Self-assessed socioeconomic status

Self-assessed socioeconomic status was measured using the MacArthur Scale of Subjective Social Status [24]. The participants were asked to place an *X* on a ladder representing their perceived socioeconomic status (in relation to income, education and prestigious jobs) in the Danish society. The ladder had ten steps, which were coded into points ranging from 1 to 10 points. The higher points the higher socioeconomic status.

### Individual factors

#### Self-rated health

Self-rated health was measured using a single item from SF-36 on general health [25]. In this study, the four response categories were dichotomised into two groups: *Excellent/very good* or *good/less good/poor.*

#### Coping

Coping was measured using six subscales of two items each from the Brief COPE Scale [26]. Each item had four response categories with a possible score between one and four. To simplify the analyses, the items from the subscales *active coping, planning and positive reframing* were grouped into the *active* coping scale. The items from the subscales *self-distraction*, *substance use* and *behavioural disengagement* were used to form the *avoidance* coping scale. Both scales were created by taking the mean of the item scores, corresponding to scores between one and six. Higher scores indicated higher levels of the coping type.

### Statistics

#### Initial analyses

Descriptive analyses were conducted to identify the main initial characteristics of the participants (*N* = 1640). Univariate analyses were performed to describe the association between the potential confounders and perceived stress (Table 2) and LMP (Table 3, Model I). The associations were tested using Fisher’s exact test (categorical variables) and the Kruskal-Wallis equality-of-populations rank test (continuous and ordinal variables).

#### Main analyses

Crude and adjusted associations between levels of perceived stress and future passive LMP were estimated using logistic regression analysis, stratified on gender, and reported as odds ratios (OR) with 95% confidence intervals (CI). The associations were tested using test for trend. Gender differences were tested by comparing the slopes from the test for trend analysis. P-values less than 0.05 were defined as statistically significant.

The adjusted analyses were carried out in two steps: First SE factors were added (Table 3, Model II) and finally individual factors were included (Table 3, Model III). Calculations were performed using the STATA statistical package (version 12.0; Stata, College Station, TX, USA).

## Results

### Initial results

Initial characteristics of the sample and LMP at follow-up, distributed on gender, are presented in Table 1. A higher percentage of women perceiving medium (48.9%) or high (7.7%) level of stress compared to men (47.5% or 4.3%). At follow-up a total of 569 (34.7%) participants had passive LMP, significant more women (37.9%) than men (30.3%).

**Table 1:** Basic characteristics and labour market participation of the study population

	Women *N* = 944	Men *N* = 696	Total *N* = 1640	p, Gender difference
	*n* (%) or mean (SD)	*n* (%) or mean (SD)	*n* (%) or mean (SD)	
LMP^a^ 2011/2012				0.001^d^
Active (%)	586 (62.1)	485 (69.7)	1071 (65.3)	
Passive (%)	358 (37.9)	211 (30.3)	569 (34.7)	
Perceived stress				0.002^d^
Low (%)	409 (43.3)	348 (50.0)	757 (46.2)	
Medium (%)	462 (48.9)	318 (45.7)	780 (47.5)	
High (%)	73 (7.7)	30 (4.3)	103 (6.3)	
Ethnicity				0.76^d^
Danish (%)	911 (96.4)	673 (96.8)	1584 (96.6)	
Western (%)	9 (1.0)	4 (0.6)	13 (0.8)	
Non-Western (%)	25 (2.6)	18 (3.6)	43 (2.6)	
Household (parents’) income				0.663^d^
Lowest (%)	150 (15.9)	102 (14.7)	252 (15.4)	
Medium (%)	540 (57.2)	395 (56.7)	935 (57)	
Highest (%)	254 (26.9)	199 (28.6)	453 (27.6)	
Secondary Education				<0.001^d^
Completed (%)	743 (78.7)	464 (66.7)	1207 (73.6)	
Not completed (%)	201 (21.3)	232 (33.3)	433 (26.4)	
LMP^a^ in 2004				0.758^d^
Active (%)	752 (79.7)	550 (79.0)	1302 (79.4)	
Passive (%)	192 (20.3)	146 (21.0)	338	
Self-assessed socioeconomic status^b^, mean (SD)	5.6 (1.6)	5.9 (1.7)	5.7 (1.7)	<0.001^e^
Self-rated health				<0.001^d^
Excellent/very good (%)	523 (55.4)	446 (64.1)	969 (59.1)	
Good/less good/bad (%)	421 (44.6)	250 (35.9)	671 (40.9)	
Active coping strategy^c^, mean (SD)	2.8 (0.5)	2.9 (0.5)	2.8 (0.5)	0.064^e^
Avoidance coping strategy^c^, mean (SD)	1.6 (0.4)	1.6 (0.4)	1.6 (0.4)	0.986^e^

*SD* standard deviation, ^a^ *LMP* labour market participation, ^b^Scale from 0 to 10;higher = better, ^c^Scale from 0 to 6;higher = more, ^d^ = Fishers’ exact test, ^e^ = Kruskal-Wallis equality-of-populations rank test

A statistically significant difference between each of the potential confounders and the level of perceived stress was found (Table 2). Secondary education completion, LMP in 2004, self-assessed socioeconomic status, self-rated health and avoidance coping were statistically significant associated with future passive LMP among women. Secondary education completion, self-assessed socioeconomic status and self-rated health were statistically significant associated with future passive LMP among men (Table 3, Model I).

**Table 2:** Basic characteristics divided on the levels of perceived stress

	Perceived stress *N* = 1640			
	Low *N* = 757	Medium *N* = 780	High *N* = 103	*p*
	*n* (%) or mean (SD)	*n* (%) or mean (SD)	*n* (%) or mean (SD)	
Gender				0.002^d^
Female (%)	409 (54.0)	462 (59.2)	73 (70.9)	
Male (%)	348 (46.0)	318 (40.8)	30 (29.1)	
Household (parents’) income				0.004^d^
Lowest (%)	95 (12.6)	130 (16.7)	27 (26.2)	
Medium (%)	437 (57.7)	447 (57.3)	51 (49.5)	
Highest (%)	225 (29.7)	203 (26.0)	25 (24.3)	
Secondary Education				<0.001^d^
Completed (%)	603 (79.7)	543 (69.6)	61 (59.2)	
Not completed (%)	154 (20.3)	237 (30.4)	42 (40.8)	
LMP^a^ in 2004				<0.001^d^
Active (%)	633 (83.6)	605 (77.6)	64 (62.1)	
Passive (%)	124 (16.4)	175 (22.4)	37 (37.9)	
Self-assessed socioeconomic status^b^, mean (SD)	6.2 (1.5)	5.5 (1.6)	4.3 (1.9)	<0.001^e^
Self-rated health				<0.001^d^
Excellent/very good (%)	562 (74.2)	386 (49.5)	21 (20.4)	
Good/less good/bad (%)	195 (25.8)	394 (50.5)	82 (79.6)	
Active coping strategy^c^, mean (SD)	3.0 (0.5)	2.7 (0.5)	2.5 (0.5)	<0.001^e^
Avoidance coping strategy^c^, mean (SD)	1.5 (0.3)	1.7 (0.4)	2 (0.5)	<0.001^e^

*SD* standard deviation, ^a^ *LMP* labour market participation, ^b^Scale from 0 to 10;higher = better, ^c^Scale from 0 to 6;higher = more, ^d^ = Fishers’ exact test, ^e^ = Kruskal-Wallis equality-of-populations rank test

**Table 3:** Logistic regressions-analysis

	Odds ratios for passive labour market participation																	
	Women									Men								
	Model I			Model II			Model III			Model I			Model II			Model III		
	Crude (*N* = 944)			Adjusted for SE factors (*N* = 944)			Adjusted for all factors (*N* = 944)			Crude (*N* = 696)			Adjusted for SE factors (*N* = 696)			Adjusted for all factors (*N* = 696)		
	OR	[95% CI]	*p*	OR	[95% CI]	*p*	OR	[95% CI]	*p*	OR	[95% CI]	*p*	OR	[95% CI]	*p*	OR	[95% CI]	*p*
Perceived stress			0.001^d^			0.035^d^			0.335^d^			0.732^d^			0.252^d^			0.045^d^
Low	1			1			1			1			1			1		
Medium	1.34	1.02–1.77		1.23	0.92–1.65		1.10	0.81–1.50		0.94	0.68–1.31		0.78	0.55–1.11		0.67	0.45–0.97	
High	2.33	1.40–3.85		1.74	1.01–2.99		1.34	0.74–2.41		1.52	0.71–3.27		0.88	0.38–2.05		0.61	0.24–1.54	
Household (parents’) income			0.070			0.856			0.739			0.570			0.614			0.633
Lowest	1.48	0.98–2.24		1.04	0.67–1.62		1.07	0.68–1.66		1.07	0.63–1.82		0.78	0.44–1.38		0.79	0.44–1.40	
Medium	1.14	0.83–1.56		1.01	0.73–1.40		1.07	0.77–1.48		1.29	0.88–1.88		1.15	0.78–1.69		1.13	0.76–1.67	
Highest	1			1			1			1			1			1		
Secondary education																		
Completed	1			1			1			1			1			1		
Not completed	2.75	2.00–3.78	<0.001	2.43	1.74–3.39	<0.001	2.4	1.71–3.36	<0.001	1.6	1.14–2.24	0.006	1.47	1.02–2.11	0.4	1.43	0.99–2.07	0.056
LMP^a^ in 2004																		
Active	1			1			1			1			1			1		
Passive	1.97	1.43–2.72	<0.001	1.72	1.23–2.40	0.002	1.74	1.24–2.44	0.001	1.07	0.72–1.59	0.725	0.89	0.57–1.38	0.599	0.87	0.56–1.35	0.524
Self-assessed socioeconomic status^b^, pr unit	0.9	0.83–0.98	0.016	0.99	0.90–1.08	0.763	1.00	0.91–1.10	0.957	0.78	0.71–0.86	<0.001	0.78	0.70–0.86	<0.001	0.79	0.71–0.88	<0.001
Self-rated health																		
Excellent/very good	1						1			1						1		
Good/less good/bad	1.42	1.09–1.85	0.009				1.21	0.91–1.62	0.195	1.51	1.08–2.10	0.015				1.38	0.96–2.00	0.082
Active coping strategy^c^, pr. unit	0.78	0.59–1.01	0.064				1.06	0.78–1.44	0.713	0.74	0.54–1.02	0.068				0.87	0.61–1.25	0.459
Avoidance coping strategy^c^, pr. unit	2.04	1.40–2.95	<0.001				1.69	1.10–2.60	0.016	1.52	0.99–2.35	0.056				1.26	0.76–2.10	0.317

^a^ *LMP* labour market participation, ^b^Scale from 0 to 10;higher = better, ^c^Scale fram 0–6;higher = more, ^d^ = test for trend

### Main results

In the crude analysis, and among women, perceived stress was significantly associated with future passive LMP (*p* = 0.001). The crude OR for passive LMP among those women perceiving medium stress was 1.34 (95% CI 1.02–1.77), and 2.33 (95% CI 1.40–3.85) among those perceiving high stress Table 3, Model I).

Among men, the crude OR for passive LMP among those perceiving medium stress was 0.94 (95% CI 0.68–1.31), and 1.52 (95% CI 0.71–3.27) among those perceiving high stress. The association was not significant (*p* = 0.732) (Table 3, Model I).

The crude association between perceived stress and future passive LMP did not differ significantly among women and men (*p* = 0.075, analysis not shown).

Among women, and after adjusting for SE factors the OR for passive LMP among those reporting medium perceived stress decreased to 1.23 (95% CI 0.92–1.65), and 1.74 (95% CI 1.01–2.99) among those reporting high perceived stress. The association remained significant (*p* = 0.035) (Table 3, Model II).

When adjusting for SE factors, the OR for passive LMP among those men reporting medium perceived stress decreased to 0.78 (95% CI 0.55–1.11), and 0.88 (95% CI 0.38–2.05) among those men reporting high perceived stress. The association was still not significant (*p* = 0.252) (Table 3, Model II).

The association between perceived stress and future passive LMP, adjusted for SE factors, differed significantly among women and men (*p* = 0.029, analysis not shown).

When adjusting for SE and individual factors, the OR for medium perceived stress decreased to 1.10 (95% CI 0.81–1.50) and for high perceived stress the OR decreased to 1.34 (95% CI 0.74–2.41) among women. The association was no longer significant (*p* = 0.335) (Table 3, Model III).

Among men, the OR for medium perceived stress decreased to 0.67 (95% CI 0.45–0.97) and for high perceived stress the OR decreased to 0.61 (95% CI 0.24–1.54) after adjusting for SE and individual factors. The association was significant (*p* = 0.045) (Table **3**, Model III).

The association between perceived stress and future passive LMP, adjusted for SE and individual factors, differed significantly among women and men (*p* = 0.029, analysis not shown).

## Discussion

The principal finding in the present study was that the association between perceived stress among 20–21 year-olds and their LMP 8 years later differed significantly among women and men, when adjusting for potential confounders (*p* = 0.029).

For women, higher levels of perceived stress increased the risk of passive LMP and for men, higher levels of perceived stress reduced the risk of passive LMP. Unlike the women (*p* = 0.335), the association was significant for men (*p* = 0.045).

The higher proportion of young women who experienced higher stress compared to young men is in accordance with findings by Glasscock et al. [14], Brooks et al. [13] (and Lesage et al. [15].

Also, both psychologically and biologically women and men tend to react differently when exposed to stress. According to Shelley E. Taylor the stress response is characterized by *fight-or-flight* in men and by *tend-and-befriend* in women [17].

Also Kunz-Ebrecht et al. found a larger awakening cortisol response on working days in women compared to men, indicating that women experience a high level of strain from family responsibilities on workdays [16]. This might help explain the observed gender difference.

Another explanation could be the way perceived stress is measured. It is possible that PSS only capture stress symptoms in women and not men. In the depression literature it is found that men manifest depression differently than women, and the original Depression Scale does not capture the depressed men. Therefore a Masculine Depression Scale has been developed [27]. Given the experiences from the Depression literature can be transmitted to stress, the findings of the present study might indicate a need of developing a Masculine PSS.

### Strengths and limitations of the study

One of the strengths of the study is the use of high-quality register data with complete follow-up. The fact that the information on outcome was obtained through a register reduces the risk of recall- and selection bias. The design was prospective, which allows an evaluation of temporal associations. Furthermore, the study extends over 8 years, which ensures that almost all of the participants have finished their education.

As perceived stress is a subjective assessment the use of self-reported questionnaire, The Perceived Stress Scale (PSS), is a reasonable method to use. PSS is designed to tap the degree to which respondents find their lives unpredictable, uncontrollable and overloading – central components of the experience of stress, and the measure takes the individual differences regarding the perception of stress into account [19].

PSS is, in its original 14 items form, correlated with negative life events, cortisol level, physical and depressive symptoms and disease, and is considered to be a valid measurement tool of stress [19, 21, 28].

When interpreting the findings of the study, the potential error induced by the 8 years follow-up, with no information about stress level or LMP of the participants should be considered. Significant things, such as disease or negative life events could have occurred during the time period and may have affected the LMP. Furthermore, according to Lazarus’s stress theory, the perceived level of stress is not a static condition and may change over time. The level of stress is influenced by daily hassles, major life events, and changes in the availability of coping [20].

The potential changes during the follow-up period can blur the association between perceived stress and the future LMP.

Regarding the measurement of perceived stress, it should be noted that because of the limited number of items in the PSS version used, the scale suffers in internal reliability and provides a less adequate approximation of the perceived stress levels compared to the 10-item and 14-item versions [19]. For this reason, non-differentiated misclassification cannot be excluded. In the future, it is recommended to use PSS with 10 or 14 items.

Not all participants returned the questionnaire (the response rate was 49%), and non-response could have affected the results. Due to missing data on perceived stress, it was not possible to determine the magnitude of the selection.

According to S. Taylor the stressed men need time off to de-stress [17]. and it is likely that the most stressed men tend not to participate. This could lead to selection bias.

Unpublished analyses of the data showed that a bigger proportion of the study population came from nuclear families with higher SE status, and a smaller proportion had another ethnicity than Danish, compared to the source population.

The presented limitations are not considered to cause serious bias in relation to the observed associations. However, caution about causal interpretation is warranted. It is likely that factors such as social support may also have an impact on perceived stress and LMP, which other studies have shown [29, 30].

Nevertheless, the associations that remained after the adjustments bear witness to a gender difference in relation to perceived stress and LMP.

The study population consisted of young adults, resident in the County of Ringkøbing, Denmark, in 2004 and very few participants had a different ethnicity than Danish. One should therefore show caution when generalizing the results to populations not similar to the study population and a future national survey is recommended.

## Conclusion

In conclusion, the analyses showed a significant gender difference in the effects of perceived stress on LMP. In the future, healthcare professionals might need to differentiate between the genders in terms of communication and prevention of health related issues. Furthermore, researchers should be aware of the gender difference and consider stratifying their analyses on gender.

From a public health point of view, it is important to understand the causes and predictors of passive LMP, in order to identify high-risk groups and developing preventive strategies. Based on this study a higher level of perceived stress does not increase the risk of future passive LMP among young men, actually it has the reverse effect. Contrary, higher levels of perceived stress tends to increase the risk of future passive LMP, among young women. In the future, more research on the area is required.
